# Supplementary material for: Baseline risk factors associated with immune related adverse events and atezolizumab
Source: Front Oncol. 2023 Feb 28;13:1138305. doi: 10.3389/fonc.2023.1138305 (PMC10011463; doi:10.3389/fonc.2023.1138305)

Suppl Fig 1: Cumulative incidence function stratified by study (panels) and treatment arm (color) to estimate the irAE probability (any grade) over time while considering death as a competing event. One separate plot for each irAE type: A) Rash, B) Hepatitis, C) Hypothyroidism, D) Hyperthyroidism, E) Pneumonitis.

A) Rash

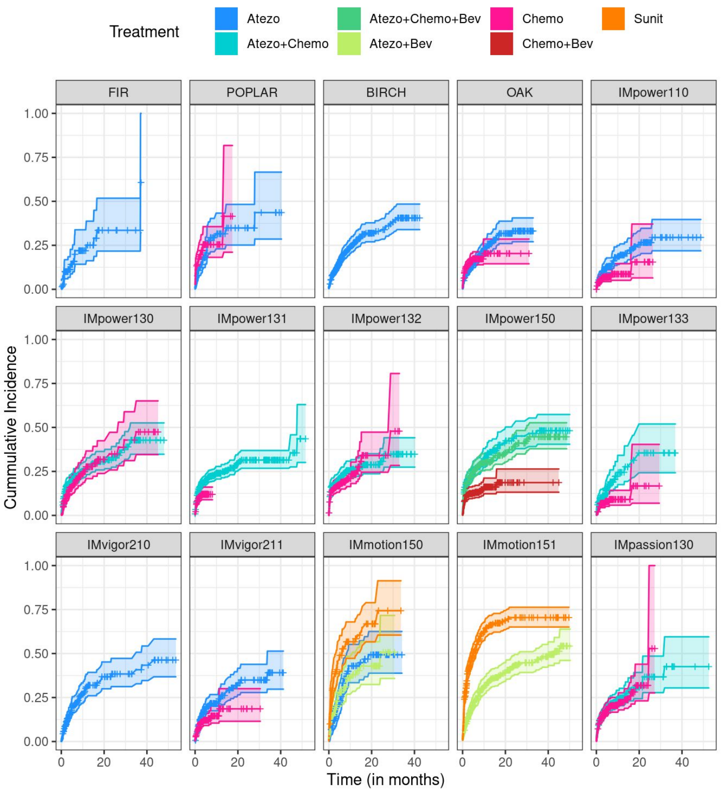

B) Hepatitis

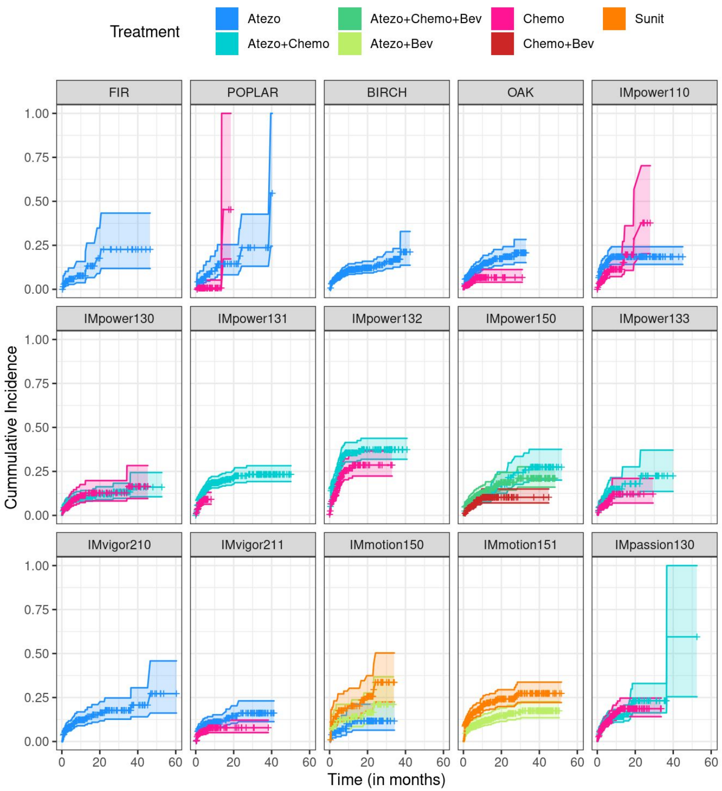

Suppl Fig 1: Cumulative incidence function stratified by study (panels) and treatment arm (color) to estimate the irAE probability (any grade) over time while considering death as a competing event. One separate plot for each irAE type: A) Rash, B) Hepatitis, C) Hypothyroidism, D) Hyperthyroidism, E) Pneumonitis.

C) Hypothyroidism

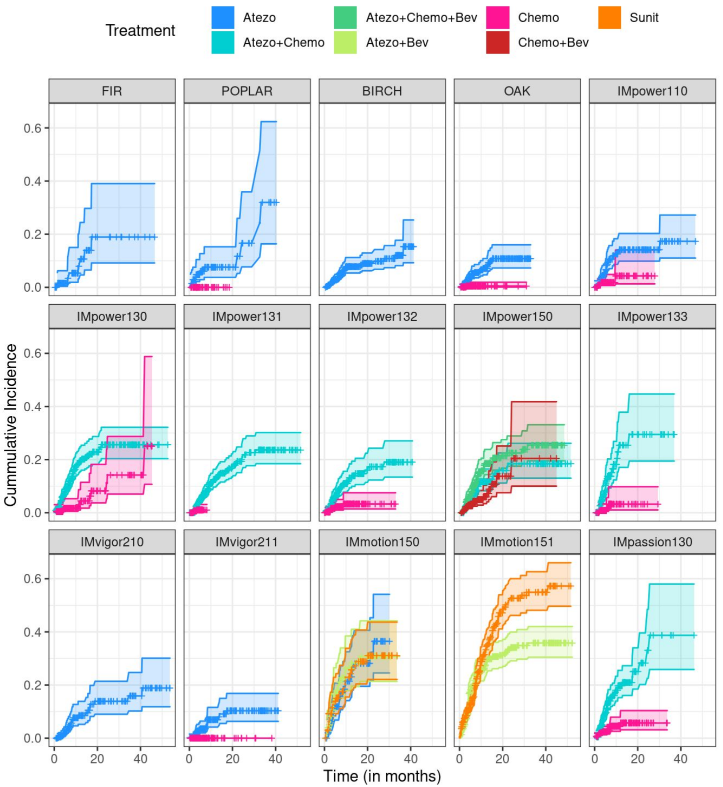

D) Hyperthyroidism

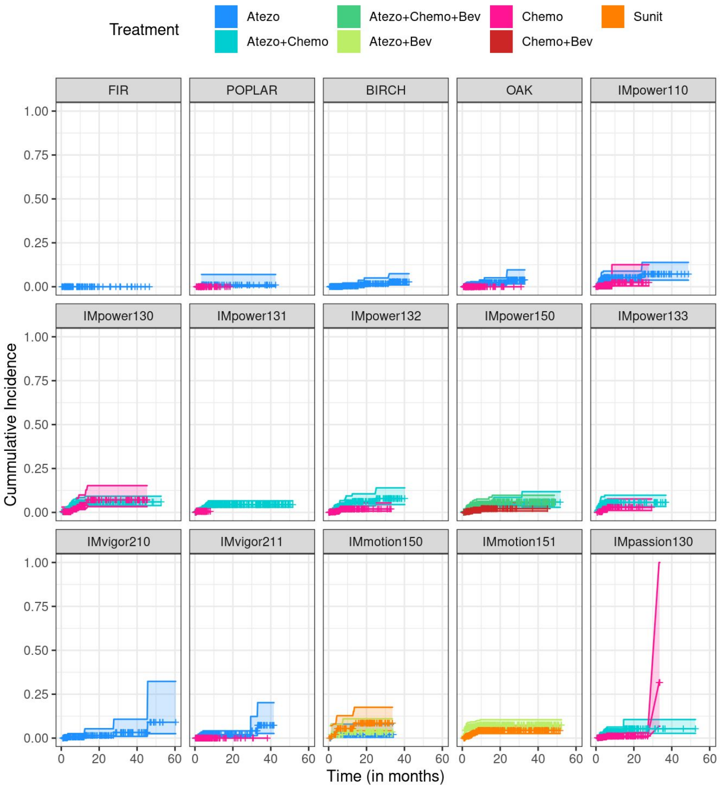

Suppl Fig 1: Cumulative incidence function stratified by study (panels) and treatment arm (color) to estimate the irAE probability (any grade) over time while considering death as a competing event. One separate plot for each irAE type: A) Rash, B) Hepatitis, C) Hypothyroidism, D) Hyperthyroidism, E) Pneumonitis.

E) Pneumonitis

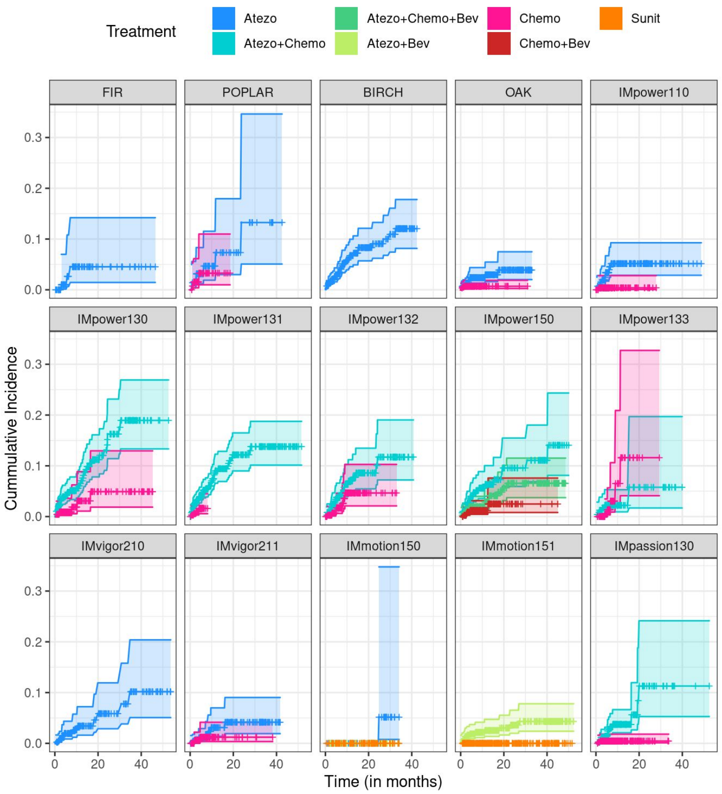

Supplement: Supplementary file 4 [file Image_1.pdf]
