# Supplementary material for: Baseline risk factors associated with immune related adverse events and atezolizumab
Source: Front Oncol. 2023 Feb 28;13:1138305. doi: 10.3389/fonc.2023.1138305 (PMC10011463; doi:10.3389/fonc.2023.1138305)

Suppl Fig 2: IPD meta-analysis results of the confounding factors age (years), sex (female vs male) and BMI (kg/m2) that were included in every risk factor model. Only confounders exhibiting a consistent and strong association with the selected irAEs of interest are shown. The baseline risk factors listed at the left side of each heatmap correspond to the IPD meta-analysis models where the confounder of interest was significant (FDR p-value < 0.05).
The color of the cells in the heatmap represents the direction of the confounder effect (red: HR>1, i.e. increased irAE risk; blue: HR<1, i.e. decreased irAE risk) and the strength of association (the darker the color the stronger the effect). The symbol within each cell indicates the significance level (FDR p-value <0.1: *, FDR p-value <0.05: **).

Association between BMI and rash

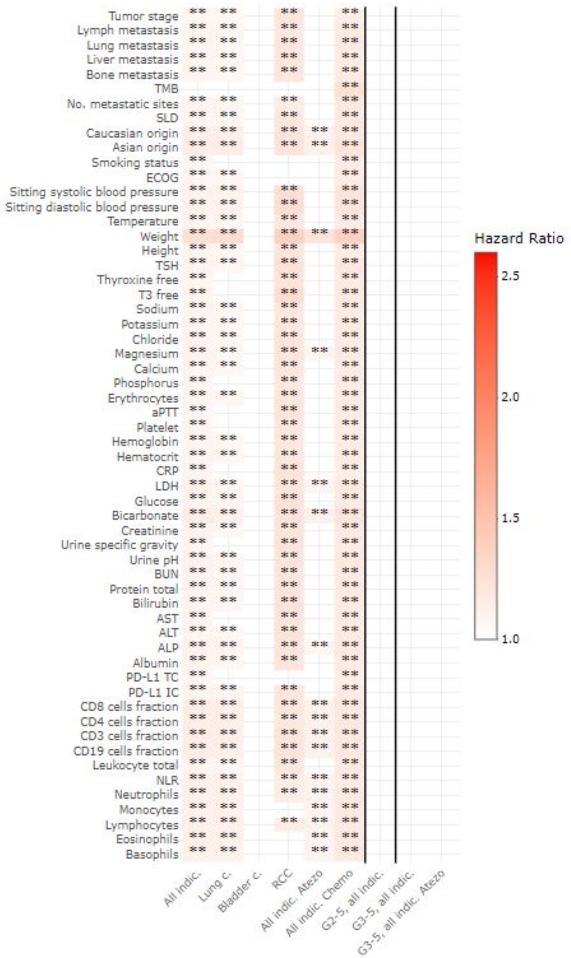

Association between age and hepatitis

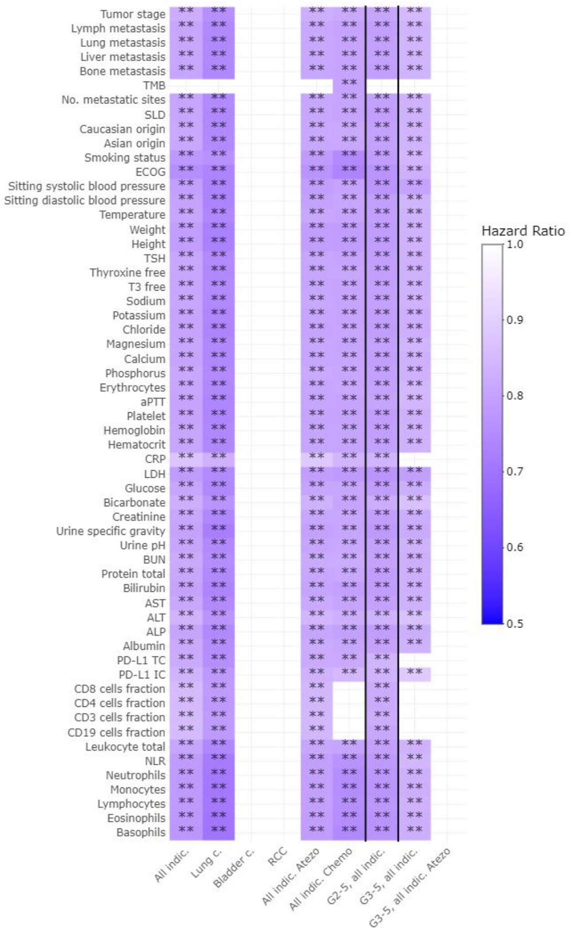

Association between sex and pneumonitis

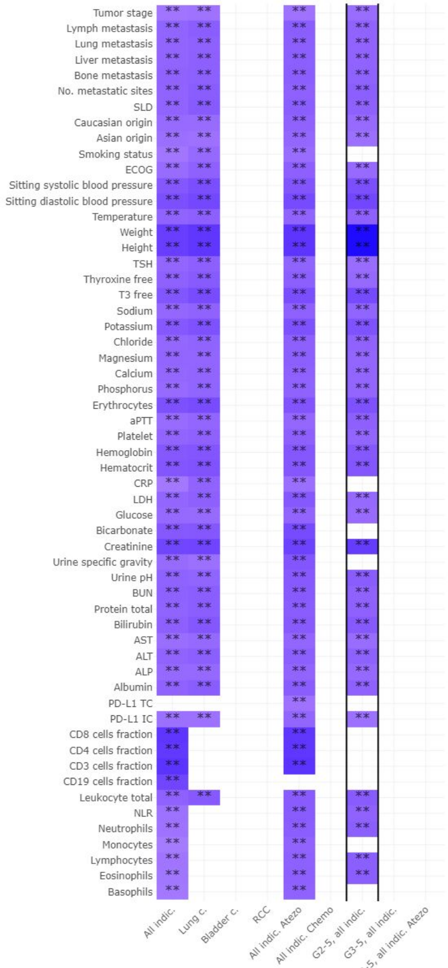

Association between sex and hypothyroidism

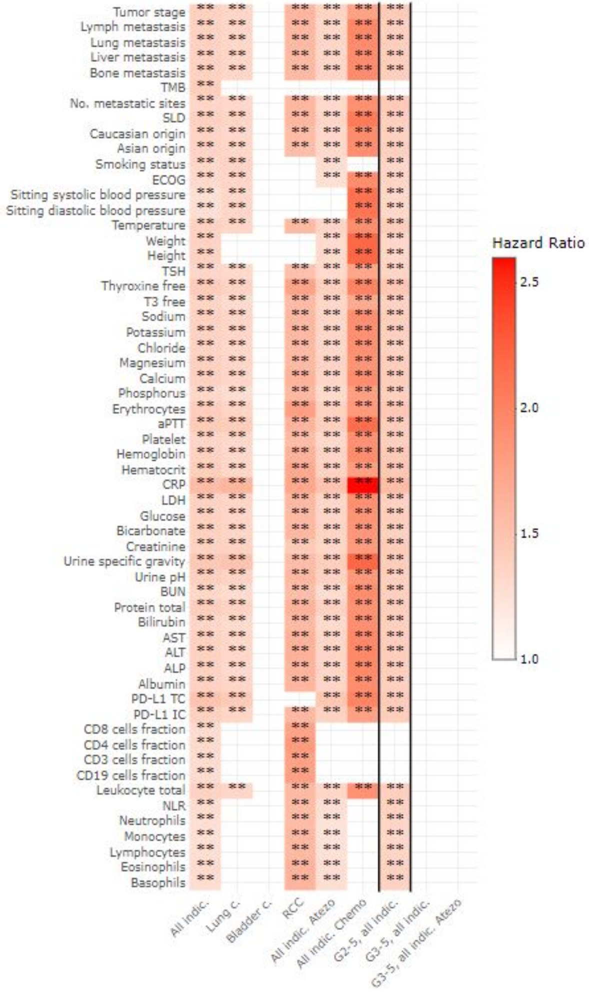

Supplement: Supplementary file 5 [file Image_2.pdf]
