# Supplementary material for: Baseline risk factors associated with immune related adverse events and atezolizumab
Source: Front Oncol. 2023 Feb 28;13:1138305. doi: 10.3389/fonc.2023.1138305 (PMC10011463; doi:10.3389/fonc.2023.1138305)

Suppl Fig 3: Comparison of the risk factors results (hazard ratios [HR] and confidence intervals [CI]) from the confounder-adjusted individual patient data meta-analysis between the atezolizumab (Atezo) and standard of care (SOC) arms. The color refers to the significance level (FDR adjusted p-values). One separate plot for each irAE type: A) Rash, B) Hepatitis, C) Pneumonitis, D) Hypothyroidism, E) Hyperthyroidism.

A) Rash B) Hepatitis C) Pneumonitis

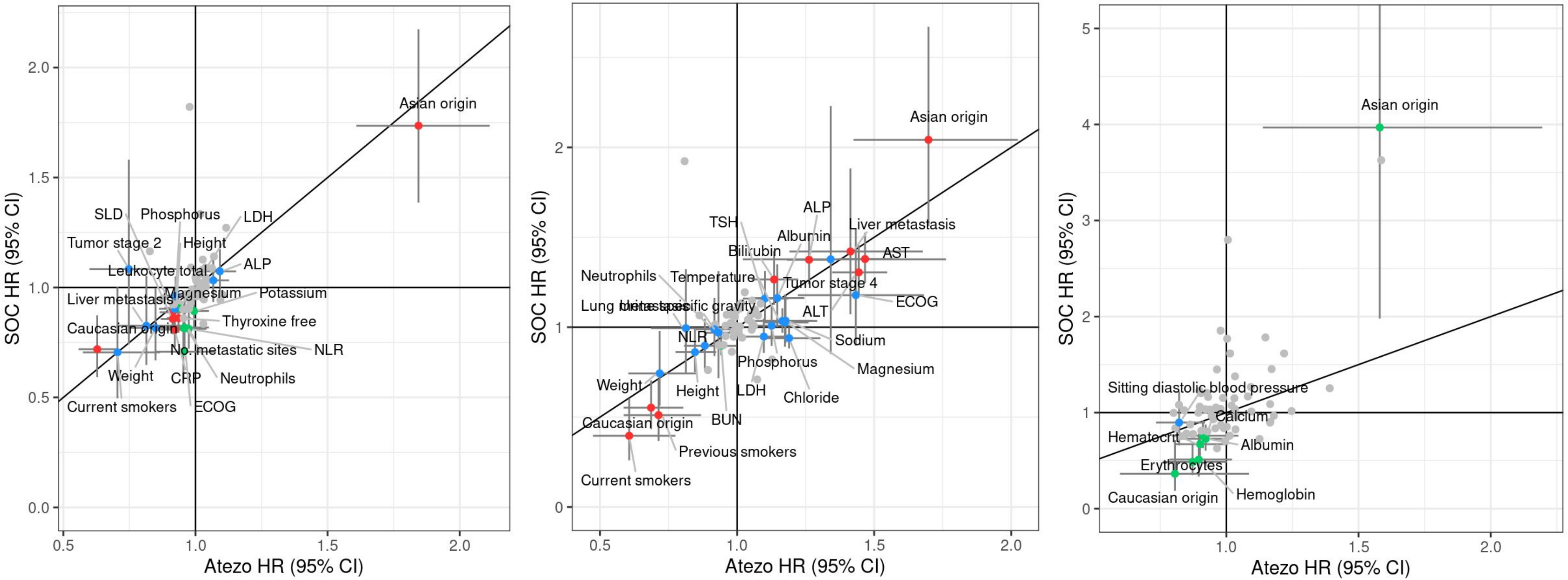

Suppl Fig 3: Comparison of the risk factors results (hazard ratios [HR] and confidence intervals [CI]) from the confounder-adjusted individual patient data meta-analysis between the atezolizumab (Atezo) and standard of care (SOC) arms. The color refers to the significance level (FDR adjusted p-values). One separate plot for each irAE type: A) Rash, B) Hepatitis, C) Pneumonitis, D) Hypothyroidism, E) Hyperthyroidism.

D) Hypothyroidism

E) Hyperthyroidism

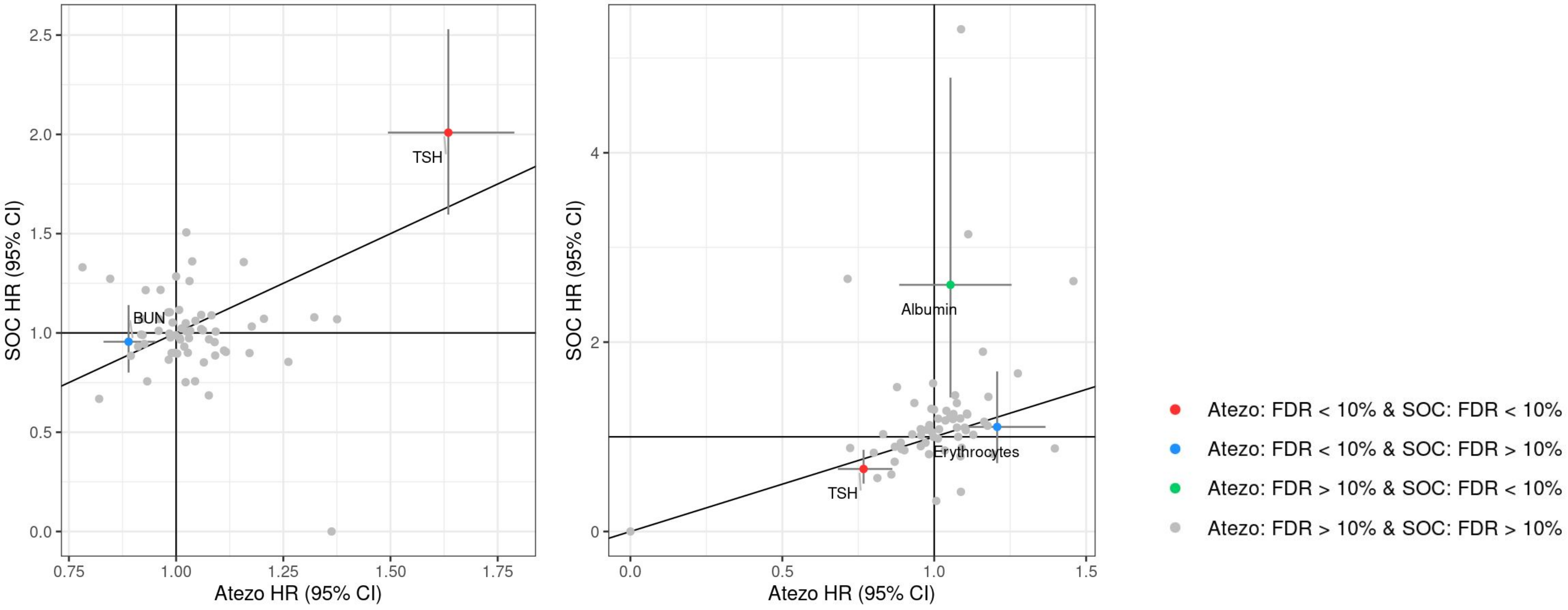

Supplement: Supplementary file 6 [file Image_3.pdf]
